# Perception of Internet Use in Relation to Health Decision-Making among Nursing Students

**DOI:** 10.3390/ejihpe13050061

**Published:** 2023-05-01

**Authors:** José A. Zafra-Agea, Noelia Calvillo-Nuñez, Òscar Gil-Jiménez, Ivan Hellín-Pijuan

**Affiliations:** 1 Gimbernat University Schools (EUG) Department of Health Sciences (Autonomous University of Barcelona, U.A.B.), Avinguda de la Generalitat, 202, 08174 Sant Cugat del Vallès, Spain; 2 Fundación Asistencial of Mutua de Terrassa, Hospital Universitario Mutua de Terrassa, Plaça del Doctor Robert nº 5, 08221 Terrassa, Spain

**Keywords:** internet addiction, social networks, internet use, health, nursing students, decision-making

## Abstract

Internet use has increased worldwide during the COVID-19 pandemic, to the point where it has inadvertently integrated into our lives. University students use the Internet daily for different purposes: seeking information, entertaining, as a teaching and learning tool, they consider social networks as a means of connection and social interaction, and to seek information to make health decisions. Because of this, the Internet and social networks have gained popularity among this group, to the point of developing an abusive use that is not perceived as an addictive risk. A descriptive analysis was performed through the adaptation of a survey about Internet use, social networks and health perception; this survey was given to nursing students of the Gimbernat School during the academic year 2021–2022. Students completed the ad hoc questionnaire (N = 486; 83.5% female, 16.3% male; only 1 declared to be non-binary gender). Our hypothesis had to do with whether the population of nursing students at Gimbernat School had increased, after the pandemic, its use of the Internet and social networks to make decisions about health problems. The objective of the study was to analyse differences in students’ habits of use of the Internet and social networks as they look for health information, their decision-making when they find the information and their perception of health as nursing students from a gender perspective. The results showed a clear positive relationship between the variables studied. Of nursing students, 60.4% spend between 20 and more than 40 h a week using the Internet, and 43.6% of these hours are spent on social networks. Of students, 31.1% make health decisions by searching for information on the Internet and consider it useful and relevant. The use of the Internet and social media in relation to health decisions is clearly affected. To try to reduce the incidence of the problem, interventions are needed regarding the prevention and/or consequences of Internet abuse and health education of student nurses as future health assets.

## 1. Summary

The use of the Internet and information technologies (ICTs) in today’s society has become indispensable for young university students, as it offers them ease and speed when searching for information and obtaining new knowledge, and allows them to make decisions about their daily lives and health [1]. Additionally, it allows them not only social interaction on a group level, but also a broad socio-cultural vision as well as intergenerational communication on a global level through social networks [2].

On the European continent, 90% of households had Internet access in 2021, according to the latest studies by the Statistical Office of the European Union (EUROSTAT 2021); access has been increasing steadily since 2013. In reference to Spain, the National Institute of Statistics estimates that there are 7.7 million family homes which have Internet access (INE 2021), of which the majority percentage, in terms of age of Internet users, corresponds to 14 and 34 years of age. In Catalonia, the percentages are very similar to the rest of the national territory. According to the statistics agency of Catalonia (IDESCAT, 2021), 100% of the population in this age range has used the Internet, especially young university students [3], 53% of whom use it for more than 3 h a day, and this has increased as a result of the increased use of asynchronous teaching–learning processes due to the COVID-19 pandemic [4,5].

Dr. Kimberly Young (1996) described Internet addiction as a disturbance of control over use that manifests itself as a set of cognitive, behavioural and physiological symptoms, characterised by preoccupations, urges or abusive behaviours that cause stress, distress and restlessness in the user, leading to distortion of the addicted person’s goals. Health refers to the balance between biological functions and their intimate interrelationship with the environment [6]. The Internet makes it possible to search for information quickly and easily to find solutions to health-related problems [7]. The most frequently consulted health-related topics are nutrition, food and healthy lifestyles (54.2% of respondents), diseases already diagnosed (52.1%), symptoms (50.9%) and the search for remedies (47%) [8]. Recent studies have shown that the greater the use of the Internet, the greater the increase in addictive behaviours, which decreases the risk perception about the information available to them when making decisions, and can lead to problematic psycho-emotional and addictive behaviours [9,10].

Social networks have become the avenue for social interaction due to the compulsory confinement that led to the modification of multiple social interactions, routines, work and education, allowing us to be in contact with the people around us without the need to share time and space [2]. The need for the network during this period has led to a false need in society to be connected to the Internet for a large part of the day, to take asynchronous classes or to telework, without fixed work or break times to satisfy social interaction needs [11].

Health risk factors associated with Internet abuse have been on the rise. An increase in symptoms such as depression, anxiety, insomnia, vision problems and/or time management problems has been reflected in their higher incidence in our population [12,13,14].

Knowing first-hand, as nursing students and future health agents, the most prevalent problems related to the use or abuse of the Internet would help us to understand how people act in their decision-making with respect to the information they find, in order to provide the most appropriate care to our population. Therefore, the aim of this study is to analyse the use of the Internet and social networks with respect to people’s health and the decision-making processes used with the information found, and whether it resolved their doubts or problems.

## 2. Data Description

### 2.1. Design and Sample

A cross-sectional survey was conducted of first-, second-, third- and fourth-year students of the university’s nursing degree program. The students were recruited during the first quarter of 2022 voluntarily: the project is part of the work of first-year students in the field of methodology and biostatistics. The inclusion criteria are the following: all first-year nursing students in the 2021–2022 academic year, enrolled in the Methodology and Biostatistics course, who wish to participate voluntarily and with prior consent. The objective of the study was to analyse the differences in the habits of use of the Internet and social networks and the search for information about students’ health and their perception of health as nursing students from a gender perspective. The first-year students decided to extend the sample voluntarily to second-, third- and fourth-year nursing students who would like to participate voluntarily and provided informed consent. These data were collected as part of their assessment, to help them learn how to analyse data from descriptive studies. Our hypothesis was based on whether the population of nursing students at Gimbernat School has increased, after the pandemic, its use the Internet and social networks to make decisions about health problems.

From a gender perspective, Internet use provides insight into changing social and cultural characteristics of women and men [15]. The sample consisted of a total of 486 nursing students from the Gimbernat University School of Barcelona (Spain), with a gender distribution of 406 women (83.5%) and 79 men (16.3%) and 1 non-binary person. The majority of respondents were in their first year of nursing (36.4%), followed by the second year (31.5%). The average age of respondents was 22.2 years; the demographic characteristics of the respondents are presented in Table 1.

### 2.2. Data Collection

This survey was conducted in the classroom upon introduction of the subject. The information was collected through a structured questionnaire with 39 points. This self-administered questionnaire was adapted and revised from a study by the Kaiser Family Foundation (Evaluation of the Use of Internet and Social Networks for Health), reviewed again, and adapted after the revision by a group of six students based on their observations. Then, the final version of the survey was obtained and the questionnaire was completed online through the Google platform [16].

Variables:

(1) Sociodemographic and health variables: age, gender, perceived health;
(2) Variables related to conditions of access and use of the Internet and social networks: frequency of use, opinion of the Internet as a source of health information, use of other sources of health information, questions about participation in social networks, motives and satisfaction;
(3) Perception of their health: questions about their perception of their health, the existence of problems at present, where they seek this help and the perception of the degree of usefulness of the Internet and social networks in relation to their need for help.

The first section also included questions about the following: if the Internet affects health, frequency of search for health information, experience of use, importance of health resources, resolution of doubts, if they think they know how to use it, health competencies, security for decision-making, if they consider themselves addicted to social networks or the Internet and evaluation and perception of their own health.

Students agreed to participate by giving their written consent at the beginning of the survey. All students were invited to participate voluntarily from the different nursing degree courses (four courses) with a total sample of 486 students.

### 2.3. Data Analysis

The statistical processing of the data was carried out with the Project R program (The R Project for Statistical Computing). The data were transferred from the online electronic questionnaire to Excel and then exported to Project R. The variables were analysed by descriptive analysis, showing the distribution using frequency and percentage counts for variables and categorical means. To deepen the analysis, the association between variables was explored. The data were encoded and quantified. Students agreed to participate by giving their written consent at the beginning of the course and in the survey.

### 2.4. Ethics

The study procedures were carried out in accordance with the Declaration of Helsinki. All procedures were approved by the Institutional Management of Gimbernat University Schools (February 2022).

## 3. Results

From the results of the survey of nursing students, we observe that the predominant gender was female, with 83.5% of the students, and the great majority of those surveyed were in the first year of nursing, with 36.4%. Meanwhile, there was a total percentage of 16.3% of men and one person of non-binary gender.

Adding the percentages, regarding the question of total hours connected to the Internet, and understanding abuse to be defined as being online at least 20 h a week either to search for information or to participate in social networks, 60.4% of the nursing students abused the Internet, dedicating between 20 and more than 40 h a week to it. From a gender perspective, there is a minimal difference between women, 60.7% of whom abused the Internet, compared to men, 59.5% of whom did. Regarding the use of social networks per week, we found that 43.6% of the students spent more than 20 h on social networks, with a difference between women (42.4%) and men (35.5%). The non-binary student remained connected to the Internet in a range of 10–20 h, whereas that time increased by almost two times when referring to connection to social networks (from 30 to 40 h) (Table 2).

Regarding messaging, 42.4% of women were online more than 20 h per week and 7.9% were online more than 40 h per week. Men spent fewer hours online; 35.5% of respondents were online more than 20 h per week, whereas 5.1% were online more than 40 h per week. As for the student of non-binary gender, its use was in the range of 20 to 30 h per week to exchange messages (Table 3).

Of the total number of students surveyed, 31.1% responded thatsay they make health decisions when they search for information on the internetInternet because they consider it relevant and, useful, and that they know how to find the information and its source easily.

Of the nursing students, 49.4% considered themselves to have the basic skills needed to be able to carry out a good health information search, whereas 35.2% were not sure if they have these competencies, and only 15.4% did not think they have the necessary competencies to search for health information when they need it.

Of the students, 62.3% responded that they know how to use the health information they find on the Internet, but 37.7% are not sure how to use it, and only 31.1% feel confident about the information they find.

Of students, 82.1% responded that they make decisions about health using the information they find on the Internet, and 62.3% resolve their doubts about their decisions based on the information they find. Of nursing students, 70.4% consider the Internet to be useful for finding information (Table 4).

Of the nursing students, 96% admitted that at least once a year they search for information on health issues on the Internet. The frequency varies; 25.5% of students searched for information on health issues a few times a year, but the percentage increased with reference to monthly searches (42.2%). Weekly searches were fewer, at 28.6%. Finally, a smaller percentage of students (3.3%) searched for health information every day. Only 0.4% of students never search for health information on the Internet.

**Figure 1:**
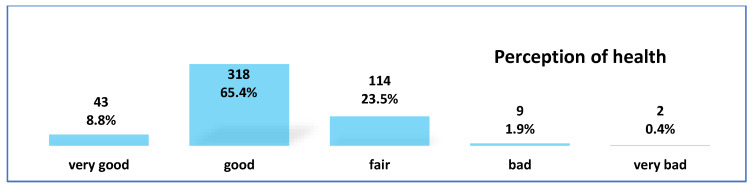
Perception of health of students.

Participating students rated their health status using the Likert scale, with five options from very good health to very bad in the previous 6 months. Very good health was reported by 8.8% of participants, 65.4% considered it good, 23.5% considered it fair and only 2.3% recognized poor health. Of students, 96% recognized the Internet as a resource to find information about health problems, and 64.6% claimed to find the right information to make health decisions (Figure 1).

In order to deepen our understanding of whether nursing students made proper use of the Internet and social media when searching for information about health problems and making decisions about their health based on information found on the Internet, and whether there was an association with their university education, a correlation analysis was performed considering age and gender. Regarding age and Internet use, we found a positive association of the contingency coefficient, value (0.50), and (0.55) of association in the use of social networks, Phi. 66. Students used this information on the Internet and social media to make decisions about their own health, resulting in a correlation, value (0.37).

Regarding making health decisions using information found on the Internet, from a gender and age perspective, we found a positive value of (0.20) and (0.52); the correlation is with the frequency of seeking information on health issues. Regarding age, there is a very positive association of (0.48). Regarding whether they use the information found to make decisions about health problems by age, we found a positive association (0.40). If they make this information available for decision-making we find a positive association (0.10).

## 4. Discussion

The objective of the study was to analyse differences in Internet and social media usage habits and the search for health information, decision-making when they find the information and the self-perception of health of nursing students, from a gender perspective.

First of all, our results show how the Internet and social networks as a source of information and communication are areas in constant expansion among young university students, and how it is a great tool for accessing information. This increase in the use of the Internet has been exponential since the COVID-19 pandemic, and especially through the use of mobile devices, as evidenced in the present study and in the consulted literature [1,17,18].

These results open a small window through which we can continue to study the s of Spanish university students on their use of the Internet and social networks, how they relate to it and how they use it when they have a problem related to their own health or when they have to make a health consultation. The results evidence that young people use the Internet mainly for leisure activities, to search for information, when they have health problems and for communication with their peers.

According to our results, our university students showed this increase in Internet use, since 60.3% of them are connected between 20 and 40 h a week to the Internet, and 43.6% to social networks, which is greater than the Spanish average when we refer to addiction, defined as being online more than 20 h a week. According to our bibliography, the average use of the Internet is 18 h 12 min per week through mobile devices, and the average use of social networks is a total of 15 h 75 min [19]. This may be due to the fact that nowadays we live connected to the Internet, whether to work, study [20] or attend meetings or classes online. In addition, many people use platforms such as YouTube, Twitch, Netflix, HBO Max, Disney +, etc. as entertainment or learning tools [21,22].

As an interesting fact, we found that the gender that uses messaging the most is female, with 42.4% of the women. Of the same gender, 7.9% work more than 40 h a week, echoing data found in the literature. The gender factor is a determinant that acts when people communicate to express their emotions and feelings through communication between equals, and is a form of liberation [23,24].

Our students show a perception of their own health as good, including very good health at 74.2%; on the other hand, 23.5% consider their health to be fair, while only 2.3% consider themselves to be in poor health. This indicates that the vast majority of students consider themselves to be in good or fair health, and that is why they generally seek information themselves on health problems and prefer the Internet to personal consultations, prioritizing its content over other traditional references [25], both from their peers and from people with specific training. In addition, they largely dump the information they find on social networks, which would be interesting to address in other studies to see what impact it could have.

As future nurses, students can awaken a critical distance from perception to reality when they start to have health problems from the use and abuse of the Internet, relate health problems to its misuse, and solve emotional problems due to mismanagement, using online literacy strategies to help with the search for and confidence in health information on social networks [26].

According to our results, 96% of students search for information on health, with 28.6% engaging in weekly searches. A total of 62.3% agree with the information provided by the Internet and its relevance to health decision-making. Regarding its application, they believe they have sufficient skills and abilities, and may not consider whether the source is adequate, and the information is confirmed. Students and future nurses could be exposed to inappropriate content, which can influence their behaviour and condition their knowledge, entailing a risk, because they are future active agents in health and health promotion/prevention [27]. Therefore, teaching students during their training about the correct use of information sources would be a key element to optimize information, knowledge, and resources when it comes to digital literacy [7].

New research is needed to allow us to know the risks that the influence of information obtained on behavioural changes can entail and whether the educational interventions currently being carried out are adequate to achieve a more critical approach to its use. It is also necessary to design new educational strategies that favour this critical analysis and digital skills [28] within the classroom, and to teach technological literacy for an improvement in search strategies to improve knowledge of health. The nurse can become a key professional in health education, and a key and strategic player in public health and health policies [19].

## 5. Limitations

Study limitations include those derived from the use of cross-sectional data. It is well known that this type of data does not allow associations or causal effects. In addition, some groups may be under-represented, and by focusing on a specific region in Spain, the results may not be generalizable. That is why it is important for future research to look at other cultural contexts and perform in-depth analyses to identify the main determinants of excessive and problematic Internet use behaviours for health decision-making, both professional and personal.

## 6. Conclusions

In this study we analysed the time nursing students spend using the Internet and social networks, their perception of health and the resources they use when searching for information to solve health problems. Students are exposed to ICT on a daily basis, which makes them vulnerable when it comes to making decisions about their health and knowing the resources available, the usefulness of these resources and whether they solve their health problems.

Regarding their sense of security about their decision-making about their health, they feel insecure about whether this information will be useful for their health. This creates a space for critical thinking about the information found and makes them think as nurses to confirm this information., As they acquire more training in the health field, they should apply more criticism only when making decisions about the information found on the Internet or social networks.

The students are unaware of their addiction to the Internet, despite positioning the Internet as their main tool of daily use, although they do recognise that it can affect their own health.

Over the last few years, the use of the Internet has increased, not only when searching for information, but also as a tool for communication, leisure, study, and work.

This lack of awareness is a problem that must be tackled through health education to prevent future problems related to the use and/or abuse of the Internet. These nursing students are future active health agents and can provide information regarding the promotion of health and prevention of disease, both in their social and work environments, and help other generations to control the proper use of the Internet and social networks.

## Figures and Tables

**Table 1:** Demographic characteristics.

Variables	Responses	*n*	%
Gender (N = 486)	Man	80	83.5%
	Woman	405	16.3%
	Non-Binary	1	0.2%
Course nursing (N = 486)	1st nursing	177	36.4%
	2nd nursing	153	31.5%
	3rd nursing	128	26.3%
	4th nursing	28	5.8%

**Table 2:** Nursing students’ time spent connected to the Internet and social networks, per week.

Connection to the Internet (Hours per Week)	Females *n* = 406 (83.5%)	Total % (83.5%)	Males *n* = 79 (16.3%)	Total % (16.3%)	Non- Binary *n* = 1 (0.2%)	Total Percentage	Rate of Validity	Cumulative Percentage
<1 h/week	3 (0.7%)	0.6%	-	-	-	0.6%	0.6%	0.6%
1 h–5 h	29 (7.1%)	6.0%	2 (2.5%)	0.4%	-	6.4%	6.4%	7%
5 h–10 h	46 (11.3%)	9.5%	8 (10.2%)	1.6%	-	11.1%	11.1%	18.1%
10 h–20 h	82 (20.2%)	16.9%	22 (27.8%)	4.5%	1 (100%)	21.6%	21.6%	39.7%
20 h–30 h	116 (28.7%)	23.9%	22 (27.8%)	4.5%	-	28.4%	28.4%	68.1%
30 h–40 h	88 (21.7%)	18.1%	16 (20.3%)	3.3%	-	21.4%	21.4%	89.5%
>40 h	42 (10.3%)	8.6%	9 (11.4%)	1.9%	-	10.5%	10.5%	100%
total	486					100%		
Connection to social networks (hours per week)								
<1 h/week	8 (2%)	1.6%	2 (2.5%)	.4%		0.6%	0.6%	0.6%
1 h–5 h	56 (13.8%)	11.5%	13 (16.5%)	2.7%	-	8.8%	8.8%	9.4%
5 h–10 h	82 (20.2%)	16.9%	17 (21.5%)	3.5%	-	20.0%	20.0%	29.4%
10 h–20 h	88 (21.7%)	18.1%	19 (24.1%)	3.9%	-	27.0%	27.0%	56.4%
20 h–30 h	75 (18.5%)	15.4%	17 (21.5%)	3.5%	-	23.7%	23.7%	80.1%
30 h–40 h	65 (16.0%)	13.4%	7 (8.9%)	1.4%	1 (100%)	16%	16%	96.1%
>40 h	32 (7.9%)	6.6%	4 (5.1%)	.8%	-	3.9%	3.9%	100.0%
total	486					100%		

**Table 3:** Internet connection time spent messaging (hours per week).

Connection Time Spent Messaging (Hours per Week)	Females *n* = 406 (83.5%)	Total % (83.5%)	Males *n* = 79 (16.3%)	Total % (16.3%)	Non-Binary *n* = 1 (0.2%)	Total Percentage	Rate of Validity	Cumulative Percentage
<1 h/week	8 (2%)	1.6%	2 (2.5%)	0.4%		0.6%	0.6%	0.6%
1 h–5 h	56 (13.8%)	11.5%	13 (16.5%)	2.7%	-	8.8%	8.8%	9.4%
5 h–10 h	82 (20.2%)	16.9%	17 (21.5%)	3.5%	-	20.0%	20.0%	29.4%
10 h–20 h	88 (21.7%)	18.1%	19 (24.1%)	3.9%	-	27.0%	27.0%	56.4%
20 h–30 h	75 (18.5%)	15.4%	17 (21.5%)	3.5%	-	23.7%	23.7%	80.1%
30 h–40 h	65 (16.0%)	13.4%	7 (8.9%)	1.4%	1 (100%)	16%	16%	96.1%
>40 h	32 (7.9%)	6.6%	4 (5.1%)	0.8%	-	3.9%	3.9%	100.0%
total	486					100%		

**Table 4:** Frequency of searching for health information on the Internet and whether this information provides confidence when making health decisions.

Security about Health Decisions Made Using the Internet	Females *n* = 406 (83.5%)	Total % (83.5%)	Males *n* = 79 (16.3%)	Total % (16.3%)	Non- Binary *n* = 1 (0.2%)	Total Percentage	Rate of Validity	Cumulative Percentage
in agreement	106 (26.1%)	21.8%	34 (43.0%)	7.0%	-	28.8%	28.8%	28.8%
disagree	101(24.9%)	20.8%	9 (11.4%)	1.9%	-	22.8%	22.8%	51.6%
undecided/not sure	158 (38.9%)	32.5%	25 (31.6%)	5.1%	-	37.7%	37.7%	89.3%
strongly agree	7 (1.7%)	1.4%	4 (5.1%)	0.8%	1 (100%)	2.3%	2.3%	91.6%
strongly disagree	34 (8.4%)	7.0%	7 (8.9%)	1.4%	-	8.4%	8.4%	100%
Total	486					100%		
frequency of searching for health information on the Internet								
every day	14 (3.4%)	2.9%	2 (2.5%)	0.4%	-	3.3%	3.3%	3.3%
never	1 (0.2%)	0.2%	1 (1.3%)	0.2%	-	0.4%	0.4%	3.7%
sometimes, weekly	116 (28.6%)	23.9%	23 (29.1%)	4.7%	-	28.6%	28.6%	32.3%
sometimes, monthly	174 (42.9%)	35.8%	30 (38.0%)	6.2%	-	42.2%	42.2%	74.5%
sometimes, annually	101 (24.9%)	20.8%	23 (29.1%)	4.7%	-	25.5%	25.5%	100%
Total	486					100%		

## Data Availability

The data could be requested by the scientific community in the ethical terms to be determined.

## References

[1] Fernández de la Iglesia J., Casal Otero L., Fernández Morante M., Cebreiro B. (2020). Actitudes y uso de Internet y redes sociales en estudiantes universitarios/as de Galicia: Implicaciones personales y sociales. Rev. Prism. Soc..

[2] Lahti H., Lyyra N., Hietajärvi L., Villber J., Paakkari L. (2021). Profiles of Internet Use and Health in Adolescence: A Person-Oriented Approach. Int. J. Environ. Res.Public Health.

[3] Garrote Rojas D., Jiménez-Fernández S., Serna Rodríguez R. (2018). Management of time and use of ICT in university students. Pixel-Bit. Rev. Medios Educ..

[4] Astobiza A. (2018). Internet y cognición social. Aníbal Monast. Astobiza.

[5] Vicente Castro F. (2021). Introducción de Psicología y crecimiento positivo. El afrontamiento maduro de la dificultad: La respuesta al COVID-19. Int. J. Dev. Educ. Psychol..

[6] Hurtado Hoyo D., Losardo R., Bianchi R. (2021). Salud plena e integral: Un concepto más amplio de salud. Rev. Asoc. Med. Argent..

[7] Blázquez Barba M., Gómez Romero D., Frontaura Fernández I., Camacho Ojeda A., Rodríguez Salas F., Toriz Cano H. (2018). Uso de Internet por los adolescentes en la búsqueda de información sanitaria. Atención Primaria.

[8] Bertomeu- Martínez M.A. (2012). Redes Sociales: Conversaciones multipantalla, riesgos y oportunidades. Tecnol. Comun. Jóvenes Promoción Salud.

[9] Masaeli N., Farhadi H. (2021). Prevalence of Internet-based addictive behaviors during COVID-19 pandemic: A systematic review. J. Addict. Dis..

[10] Kovačić Petrović Z., Peraica T., Kozarić-Kovačić D., Rojnić Palavra I. (2022). Internet use and internet-based addictive behaviours during coronavirus pandemic. Curr. Opin. Psychiatry.

[11] Odgers C.L., Jensen M.R. (2020). Adolescent development and growing divides in the digital age. Dialogues Clin. Neurosci..

[12] Aznar Díaz I., Kopecký K., Romero Rodríguez J., Cáceres Reche M., Trujillo Torres J. (2020). Patologías asociadas al uso problemático de internet. Una revisión sistemática y metaanálisis en WOS y Scopus. Investig. Vivliotecnológica.

[13] Romero-Rodríguez J., Martínez-Heredia N., Campos Soto M., Ramos Navas-Parejo M. (2021). Influencia de la Adicción a Internet en el Bienestar Personal de los Estudiantes Universitarios. Health Addict. Drog..

[14] Younes F., Halaw G., Jabbou H., El Osta N., Karam L., Hajj A. (2016). Internet Addiction and Relationships with Insomnia, Anxiety, Depression, Stress and Self-Esteem in University Students: A Cross Sectional Designed Study. PLoS ONE.

[15] Sokil J.P., Osorio L. (2022). Producción científica en el campo de los estudios de género: Análisis de revistas seleccionadas de Web of Science (2008–2018). Rev. Española Doc. Científica.

[16] Lima-Pereira P., Bermúdez-Tamayo C., Jasienska G. (2012). Use of the Internet as a source of health information amongst participants of antenatal classes. J. Clin. Nurs..

[17] Rojas-Jara C., Henríquez F., Sanhueza F., Núñez P., Inostroza E., Solís A., Contreras D. (2018). Adicción a Internet y uso de redes sociales en adolescentes: Una revisión. Rev. Española Drog..

[18] Urrunaga Ramírez J., Tello Cabrera C., Armas Marinos G. (2020). Adicción a redes sociales y rendimiento académico. Rev. Psicol..

[19] Garcia-Mendez C., García-Padilla F., Romero-Martín M., Sosa-Cordobés E., Domínguez-Pérez M.M., Robles-Romero J. (2022). Social networks: A quality tool for health dissemination?. J. Educ. Health Promot..

[20] Mangialavori S., Russo C., Jimeno M.V., Ricarte J.J., D’Urso G., Barni D., Cacioppo M. (2021). Insecure attachment styles and unbalanced family functioning as risk factors of problematic smartphone use in spanish young adults: A relative weight analysis. Eur. J. Investig. Health Psychol. Educ..

[21] Carlos O.M. (2022). The communication of the main national symphony orchestras with the arrival of COVID-19. Cult. Rev. De Gestión Cult..

[22] Instituto Nacional de Estadística (INE) Hogares que Tienen Acceso a Internet y Hogares Que Tienen Ordenador. Porcentaje de Menores Usuarios de TIC. Mujeres y Hombres en España. https://www.ine.es/ss/Satellite?L=es_ES&c=INESeccion_C&cid=1259925529799&p=%5C&pagename=ProductosYServicios%2FPYSLayout&param1=PYSDetalle&param3=1259924822888.

[23] Mari E., Biondi S., Varchetta M., Cricenti C., Fraschetti A., Pizzo A., Barchielli B., Roma P., Vilar M.M., Sala F.G. (2023). Gender differences in internet addiction: A study on variables related to its possible development. Comput. Hum. Behav. Rep..

[24] Miranda S., Trigo I., Rodrigues R., Duarte M. (2023). Addiction to social networking sites: Motivations, flow, and sense of belonging at the root of addiction. Technol. Forecast. Soc. Change.

[25] Portillo-Reyes V., Ávila Amaya J., Capps J.W. (2021). Relación del Uso de Redes Sociales con la Autoestima y la Ansiedad en Estudiantes Universitarios. Enseñanza Investig. Psicol..

[26] Freeman J.L., Caldwell P.H.Y., Scott K.M. (2022). How Adolescents Trust Health Information on Social Media: A Systematic Review. Acad. Pediatr..

[27] Herrera-Peco I. (2021). Comunicación en salud y redes sociales: Necesitamos más enfermeras Health Communication and social media: We need more nurses. Rev. Cient. Soc. Esp. Enferm. Neuro..

[28] Valkenburg P.M., Meier A., Beyens I. (2022). Social media use and its impact on adolescent mental health: An umbrella review of the evidence. Curr. Opin. Psychol..

